# ZENK Activation in the Nidopallium of Black-Capped Chickadees in Response to Both Conspecific and Heterospecific Calls

**DOI:** 10.1371/journal.pone.0100927

**Published:** 2014-06-25

**Authors:** Marc T. Avey, Laurie L. Bloomfield, Julie E. Elie, Todd M. Freeberg, Lauren M. Guillette, Marisa Hoeschele, Homan Lee, Michele K. Moscicki, Jessica L. Owens, Christopher B. Sturdy

**Affiliations:** 1 Department of Psychology, University of Alberta, Edmonton, Alberta, Canada; 2 Department of Psychology, Algoma University, Sault Ste. Marie, Ontario, Canada; 3 Department of Psychology, Helen Wills Neuroscience Institute, University of California, Berkeley, California, United States of America; 4 Departments of Psychology and Ecology & Evolutionary Biology, University of Tennessee, Knoxville, Tennessee, United States of America; 5 Department of Psychology, University of Tennessee, Knoxville, Tennessee, United States of America; UCLA, United States of America

## Abstract

Neuronal populations in the songbird nidopallium increase in activity the most to conspecific vocalizations relative to heterospecific songbird vocalizations or artificial stimuli such as tones. Here, we tested whether the difference in neural activity between conspecific and heterospecific vocalizations is due to acoustic differences or to the degree of phylogenetic relatedness of the species producing the vocalizations. To compare differences in neural responses of black-capped chickadees, *Poecile atricapillus*, to playback conditions we used a known marker for neural activity, ZENK, in the caudal medial nidopallium and caudomedial mesopallium. We used the acoustically complex *‘dee’* notes from *chick-a-dee* calls, and vocalizations from other heterospecific species similar in duration and spectral features. We tested the vocalizations from three heterospecific species (chestnut-backed chickadees, tufted titmice, and zebra finches), the vocalizations from conspecific individuals (black-capped chickadees), and reversed versions of the latter. There were no significant differences in the amount of expression between any of the groups except in the control condition, which resulted in significantly less neuronal activation. Our results suggest that, in certain cases, neuronal activity is not higher in response to conspecific than in response to heterospecific vocalizations for songbirds, but rather is sensitive to the acoustic features of the signal. Both acoustic features of the calls and the phylogenetic relationship between of the signaler and the receiver interact in the response of the nidopallium.

## Introduction

In songbirds, the caudomedial mesopallium (CMM) and caudomedial nidopallium (NCM) both display increased neuronal activation in response to conspecific vocalizations [1], [2]. Immediate early gene expression, measured using markers for ZENK (zif-268, egr-1, NGFI-A, Krox-24) [3], is higher in CMM and NCM following playback of conspecific vocalizations, relative to heterospecific vocalizations, whereas both of these stimulus classes generally elicit greater ZENK expression compared to artificial stimuli such as tones [1], [4]–[6] or silence [7]–[11]. Differences in ZENK response between conspecific and heterospecific signals may be driven by phylogenetic distance between species or by the acoustic properties of the signals, or both. Communicative signals of species often reflect common ancestry (vocal signals in anurans: [12], [13]; acoustic signals in crickets: [14]; visual display diversity and complexity in lizards: [15], [16]; but see [17] for an example of minimal phylogenetic influence in calls of African clawed frog species). Given the shared coevolutionary history of signals and signal production, we might therefore expect responsiveness for signals – and the neuroanatomical and physiological mechanisms that underlie them – to be strongly influenced by phylogeny as well. For instance, in the case of sensory biases in the anuran genera *Physalaemus* and *Engystomops*, responses to signals are similar among closely-related species even though the signals of these species are quite distinct acoustically [18], [19]. However, the level of phylogenetic divergence between signalers and the diversity of responses by receivers may not be strongly correlated. Responses to signals may be influenced primarily by the acoustic similarity between heterospecific and conspecific signals, without regard for shared ancestry [20]. This latter possibility could stem from similar selection pressures shaping signals and responses in unrelated species [17]. To address these questions, more work is needed to understand receiver behavioural and neural responses to signals of conspecific and heterospecific species [21]. Here, we assessed whether the phylogenetic similarity of species producing heterospecific vocal signals correlates with the amount of immediate early gene expression in a songbird species, or whether acoustic similarity among signals might be a better predictor of receiver neural responses.

We examined ZENK expression in black-capped chickadees (*Poecile atricapillus*), a temperate North American songbird species that belongs to the genus *Poecile*. All the members of this closely related group of species [22] use a complex call (the namesake *chick-a-dee* call [23], [24]). Studies of CMM and NCM in black-capped chickadees have revealed robust ZENK expression in response to their tonal conspecific *fee-bee* song and acoustically complex *chick-a-dee* call [25], [26]. In this study, we tested how phylogenetic and acoustic similarity influences the neural responses of CMM and NCM to conspecific and heterospecific calls in black-capped chickadees.

Our objective was to examine whether phylogenetic differences could drive the ZENK response with acoustically similar vocalizations as playback stimuli. Behavioural paradigms have shown that the ‘*dee*’ call of black-capped chickadees contains acoustic features used for species identification [27]–[30]. We identified several calls from different species that produce ‘*dee*’-like notes with similar acoustic properties (e.g. broad band, harmonic-like frequency stacks) and that also have a similar duration to black-capped chickadee ‘*dee*’ notes. We selected three species, two of which are sympatric with the black-capped chickadee in some geographic locations (but not in the regions from which we collected black-capped chickadees). Both were North American species, the chestnut-backed chickadee (*Poecile rufuscens*) [31], and tufted titmouse (*Baeolophus bicolor*) [32]. The calls of these two species share similar acoustic properties (e.g. loudest frequency of ‘*dee*’-like notes) with the black-capped chickadees. Chestnut-backed chickadee ‘*dee*’ notes are the most similar acoustically to black-capped chickadees of all chickadee species and the species is more closely related to black-capped chickadees than are tufted titmice [22]. We also selected a distantly related species as a control that produces calls (the distance call) that share similar acoustic properties to the black-capped chickadees ‘*dee*’ notes: female and male zebra finches (*Taeniopygia guttata*) [33]. If responses are driven primarily by evolutionary relatedness, we predicted that black-capped chickadees would show decreased ZENK expression when exposed to calls of distantly related species, even if the acoustic properties of the vocalizations were similar. However, if responses depend primarily on acoustic similarity, we expected that chickadees would show robust ZENK expression to all of the call variants tested here.

We first investigated the difference between Playback Condition (stimulus groups: black-capped chickadees, chestnut-backed chickadees, tufted titmice, female zebra finches, male zebra finches, and reversed black-capped chickadee calls as a control) and Brain Region (CMM, NCMd, NCMv) as measured by the mean number of cells positive for ZENK. Previous studies have found differences in the amount of ZENK expression between these brain regions which may perform different functions in auditory processing [34]. We also evaluated differences between ZENK expression in each hemisphere as a secondary outcome. Previous studies in zebra finches have found lateralization of ZENK expression in CMM and NCM with combined conspecific audio/visual presentation [35] and lesion studies have found hemispheric differences in song processing [36].

## Methods

### Ethics Statement

All studies were conducted in accordance with the Canadian Council on Animal Care Guidelines and Policies with approval from the Animal Care and Use Committee for Biosciences for the University of Alberta (Protocol number 682/12/11), and the University of Calgary Life and Environmental Sciences Animal Care Committee (BI11R-10). Chickadees were captured under an Environment Canada Scientific permit (Permit number 09-MB-SC027), Alberta Sustainable Resource Development (Fish and Wildlife Division) Collection and Research permits (Permit numbers 47908 and 47910), and a City of Edmonton Parks Permit.

### Subjects and Housing

Thirty adult male black-capped chickadees were captured via potter traps from several regions in and around Edmonton, Alberta, Canada (53^°^32′N, 113^°^29′W) and Kananaskis Country, Alberta, Canada (51^°^02′N, 115^°^03′W). Sex was initially determined by DNA analysis (Griffiths, 2000) and subsequently confirmed by post-mortem examination of reproductive organs. Prior to experimental sessions, chickadees were housed individually in cages in a colony room with a light cycle that approximated the natural weekly light cycle for Edmonton. All cages contained perches and bedding material, as well as baths and cover for the birds to hide behind for environmental enrichment. Food (small bird maintenance, Mazuri) and water (dH_2_0) were provided *ad libitum* as well as supplementation of hard-boiled eggs with spinach two times a week and meal worms three times a week were provided. Colony room temperatures were maintained at about 20°C.

### Stimuli

Birds from which call stimuli were obtained were neither used in the experiment nor housed with birds used in the experiment. We used different recording sources for each species, and recorded both males and females from each species. Black-capped chickadees were recorded at Elk Island National Park (53^°^36′N, 112^°^51′W) using a Marantz PMD670 solid-state recorder and a Sennheiser ME67 directional microphone (Saul Mineroff Electronics, Elmont, New York, USA). Chestnut-backed chickadee calls were recorded on Vancouver Island, Canada using a MiniDisc recorder (model MZ-N1, Sony Corp., Tokyo, Japan) connected to a Sennheiser omnidirectional microphone (model ME62, Sennheiser Corp., Wedemark, Germany) or from the Macaulay Library of Natural Sounds at the Cornell Laboratory of Ornithology, which consisted of recordings from many different individuals with different recording equipment. The tufted titmouse calls were recorded at field sites and in an aviary at the University of Tennessee, Knoxville using a Fostex recorder (Fostex FR-2 Field Memory Digital Recorder) and Sennheiser directional microphone (Me-66). We recorded both male and female zebra finches calls because this species' distance call is highly sexually dimorphic [33] unlike chickadee or titmice calls. Zebra finch distance calls were recorded at the University of Saint-Etienne, France in a soundproof room in cages (40×25×35 cm) using a Marantz recorder (Marantz PMD 670) and Sennheiser omni-directional microphone (Sennheiser MD42). All vocalizations were bandpass filtered between 500 Hz and 14,000 Hz in Goldwave (Goldwave, St. John's, Newfoundland & Labrador, Canada) to remove background noise and equalized using SIGNAL version 5.0 sound analysis software (Engineering Design 2003, Berkeley, CA, USA). All stimuli were 16 bit and with a 44,100 Hz sample rate.

**Figure 1:**
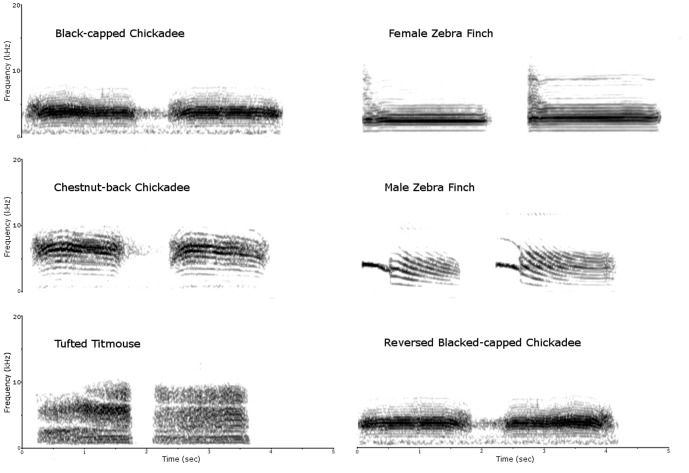
Example call spectrograms. Stimulus sets for (A) black-capped chickadee ‘*dee*’ note calls; (B) reversed black-capped chickadee ‘*dee*’ note calls; (C) female zebra finch distance calls; D) chestnut-backed chickadee ‘*dee*’ note calls; (E) tufted titmouse calls; and (F) male zebra finch distance calls (fast Fourier transform window  = 256 points).

We constructed six stimulus sets: (1) black-capped chickadee ‘*dee*’ note calls, (2) chestnut-back chickadee ‘*dee*’ note calls, (3) tufted titmouse ‘*dee*’ note calls, (4) female zebra finch distance calls, (5) male zebra finch distance calls, (6) reversed black-capped chickadee ‘*dee*’ note calls (Fig. 1.). Each stimulus set consisted of four vocalizations produced by two separate individuals of the same species (i.e. a_1_a_2_-b_1_b_2_; two different renditions of ‘*dee*’ notes or distance calls per individual). These four vocalizations were then played within a 10 s period and followed by 50 s of silence (i.e. a_1_a_2_-b_1_b_2_-silence; 60 s total) to form a 60 s sequence. Each 60 s sequence was repeated 30 times to generate a 30 min stimulus set for each species. When constructing the stimulus sets, the total duration of sound stimulation (defined as the sum of the durations of the four vocalizations of a set) was matched within 1 ms of precision between sets of all chickadee and tufted titmouse calls. The male zebra finch calls were also within ∼1 ms of duration to the matching stimulus sets of chickadees and tufted titmouse calls. However, female zebra finch calls are naturally longer than the ‘*dee*’ notes of chickadees; thus the duration of these sets were ∼250 ms longer than the other stimulus sets (see Table 1. for durations).

**Table 1:** Duration (ms) of ‘*dee*’-like Notes per Iteration for Each Stimulus Set.

Stimulus Set	Black-capped Chickadee	Reversed Black-capped Chickadee	Chestnut-backed Chickadee	Tufted Titmouse	Female Zebra Finch	Male Zebra Finch
1	643	643	643	643	850	642
2	645	645	645	645	848	646
3	626	626	626	626	847	626
4	680	680	680	679	846	681
5	664	664	664	664	855	663
Median	645	645	645	645	848	646
Maximum	680	680	680	679	855	681
Minimum	626	626	626	626	846	626

### Playback Equipment

Playbacks were recorded using an AKG C 1000S condenser microphone (frequency response: 50–20,000 Hz; AKG Acoustics, Vienna, Austria), and a solid-state recorder (Marantz PMD670, D&M Professional, Itasca, IL, USA). Stimuli were played back through a Fostex FE108Σ speaker (Fostex Corp., Japan; frequency range 80–18,000 Hz) and amplifier (Cambridge Audio A300; London, UK) with an MP3 player (Creative ZEN; Singapore). The amplitude of the playback stimuli was measured at the level of the perches from the center position of the cage and playback amplitude was set to approximately 74 dB SPL [37] using a sound-level meter (A weighting, slow response; Radio Shack 33-2055).

### Study Design and Playback Procedure

Black-capped chickadees were randomly assigned to one of six groups. The five treatment groups correspond to the stimulus sets 1–5 above and the control group to stimulus set 6 (n = 5 black-capped chickadees per set). Each individual chickadee was exposed to a stimulus set in isolation. The playbacks were conducted in a sound attenuating chamber (inner dimensions 58×168×83 cm; Industrial Acoustics Corporation, Bronx, New York, USA) where individual birds were housed overnight in a modified home cage which contained three perches at the level of the speaker. Food and water were provided *ad libitum* by two water bottles and one food cup at either end of the cage. The light cycle used in the chamber was the same as that used in the colony room and the experiment was conducted before winter solstice in December when *chick-a-dee* calling is naturally high and *fee-bee* song production is low [38], [39]. Pre-playback baseline (30 min of silence) and playback sessions (30 min) were recorded (audio and video) using bullet cameras (Swann Bullet-cam, SW-P-BCC, Swann, Santa Fe Springs, CA, USA) starting at approximately 10:00 am each day (during the normal light cycle period). Following the 30 min playback period, the chamber lights were extinguished for 1 h.

At the end of the 1 h post-playback period each bird was euthanized via an overdose (0.03 ml) of 100 mg/ml ketamine and 20 mg/ml xylazine intramuscularly (1∶1). Responsiveness was assessed via a toe pinch and eye blink before proceeding to a transcardial perfusion with heparinized 0.1 M phosphate buffered saline (PBS) followed by 4% paraformaldehyde. Following perfusion, the brain was removed and placed in 4% paraformaldehyde for 24 h, then placed in a 30% sucrose PBS solution for approximately 24 h until saturated, and then frozen in dry ice and stored at −80°C.

### Histology

For each bird, 48 40 µm sagittal sections were collected from each hemisphere. First, the brain was cut in half along the midline using a razor blade, then sections were taken using a cryostat starting from the midline and moving laterally and placed into 0.1 M PBS. We processed brains in batches randomized across treatment groups. Sections were washed for 5 min in 0.1 M PBS, incubated in 0.5% H_2_O_2_ for 15 min, and washed three times for 5 min again in 0.1 M PBS. Next, sections were incubated in 10% normal goat serum for 20 h at room temperature, followed by incubation in the primary antibody (egr-1, catalogue # sc-189, Santa Cruz Biotechnology, Santa Cruz, CA, USA) at a concentration of 1∶5,000 in 0.1 M PBS containing Triton X-100 (PBS/T) for 24 h at room temperature. Sections were then washed three times in PBS/T and incubated in biotinylated goat-antirabbit antibody (Vector Labs, Burlington, ON, Canada) for 1 h (1∶200 dilution in PBS/T). Next, sections were washed three times for 5 min in PBS/T, incubated in avidin–biotin horseradish peroxidase (ABC Vectastain Elite Kit; Vector Labs, Burlington, ON, Canada) for 1 h, and washed three times for 5 min in 0.1 M PBS. Sections were visualized using 3,3′-diaminobenzidine tetrachloride (Sigma FastDAB, D4418, Sigma–Aldrich, Santa Fe Springs, CA, USA) and mounted on gelatin-coated microscope slides. Once the sections had dried to the slides, they were further dehydrated in a graded series of ethanol, cleared with citrisolv (Fisher Scientific, Ottawa, ON, Canada), and then immediately protected with cover slips affixed with Permount (Sigma–Aldrich).

### Imaging

**Figure 2:**
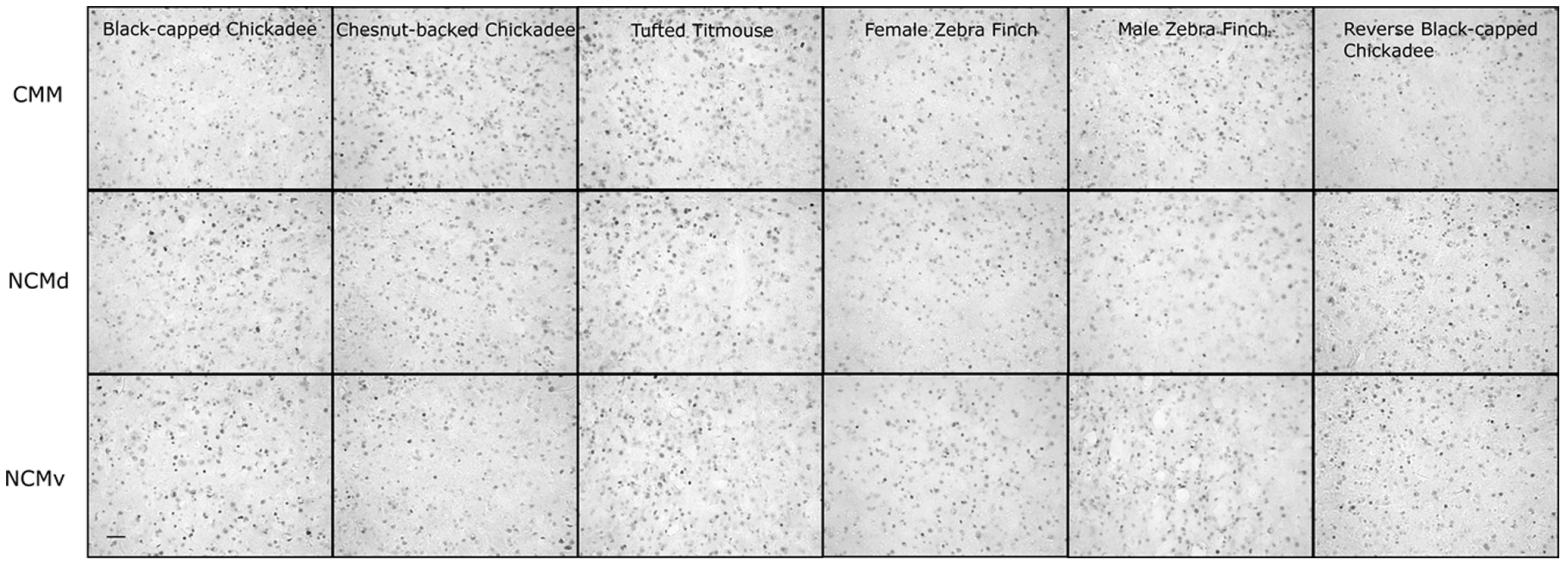
Example ZENK expression in CMM, NCMv, NCMd for each stimulus. Black-capped chickadee, chestnut-back chickadee, tufted titmouse, female zebra finch, male zebra finch, and reversed black-capped chickadee. Scale bar 50 µm.

To quantify the amount of ZENK immunoreactivity (ZENK-ir) we captured images from CMM, and the dorsal and ventral portions of NCM (Fig. 2.). Images were captured from neuroanatomical locations used previously [26], [35], [37], [40]. Briefly, CMM was defined as the most caudal area bounded by the lateral ventricle and the caudal-ventral boundary of the mesopallial lamina (LaM). NCM was defined by the lateral ventricle (dorsal, ventral, and caudal borders) and the dorsal and ventral images were captured without overlap. An observer blind to the playback condition of the bird conducted all imaging. We quantified the amount of ZENK-ir in the first eight sections of tissue for each hemisphere starting with the first section in which mesopallium was contiguous with the rostral portion of the nidopallium to ensure the orientation of the neostriatum was correct.

For each black-capped chickadee, 48 images in total were collected: 8 images for each of the three brain areas per hemisphere. Images (0.20×0.15 mm) were captured using a Leica microscope (DM 5500B; Wetzlar, Germany) with a 40× objective and a Retiga EX*i* camera (Qimaging, Surrey, British Columbia, Canada) using Openlab 5.1 (Perkin Elmer Inc., Waltham, Massachusetts, USA). The number of immunoreactive cells was counted using a semi-automated protocol in ImageJ (NIH, v.1.36b, NIH, Bethesda, Maryland, USA).

### Statistical Analysis

We conducted the statistical analysis using SPSS (IBM SPSS Statistics for Windows, Version 19.0. Armonk, NY: IBM Corp.). In brief, we conducted a repeated measures analysis of variance (ANOVA) to examine the effects of Playback Condition (stimulus groups: black-capped chickadees, chestnut-backed chickadees, tufted titmice, female zebra finches, male zebra finches, and reversed black-capped chickadees) as a between-subject factor and Brain Region (CMM, NCMv, or NCMd), Hemisphere (left and right), and Medial-Lateral Position (section numbers 1–8) as within-subject factors. We report all significant main effects and interactions.

The number of ZENK-ir cells was the dependent measure and we conducted a Tukey's post hoc analysis on Playback Condition and Bonferroni corrected pairwise comparisons on Brain Region and Hemisphere. Our alpha level for significance was set at 0.05. The number of cells per mm^2^ is given as mean over subjects (M) ± standard error of the mean (SEM; calculated using Microsoft Excel 2010).

## Results

**Figure 3:**
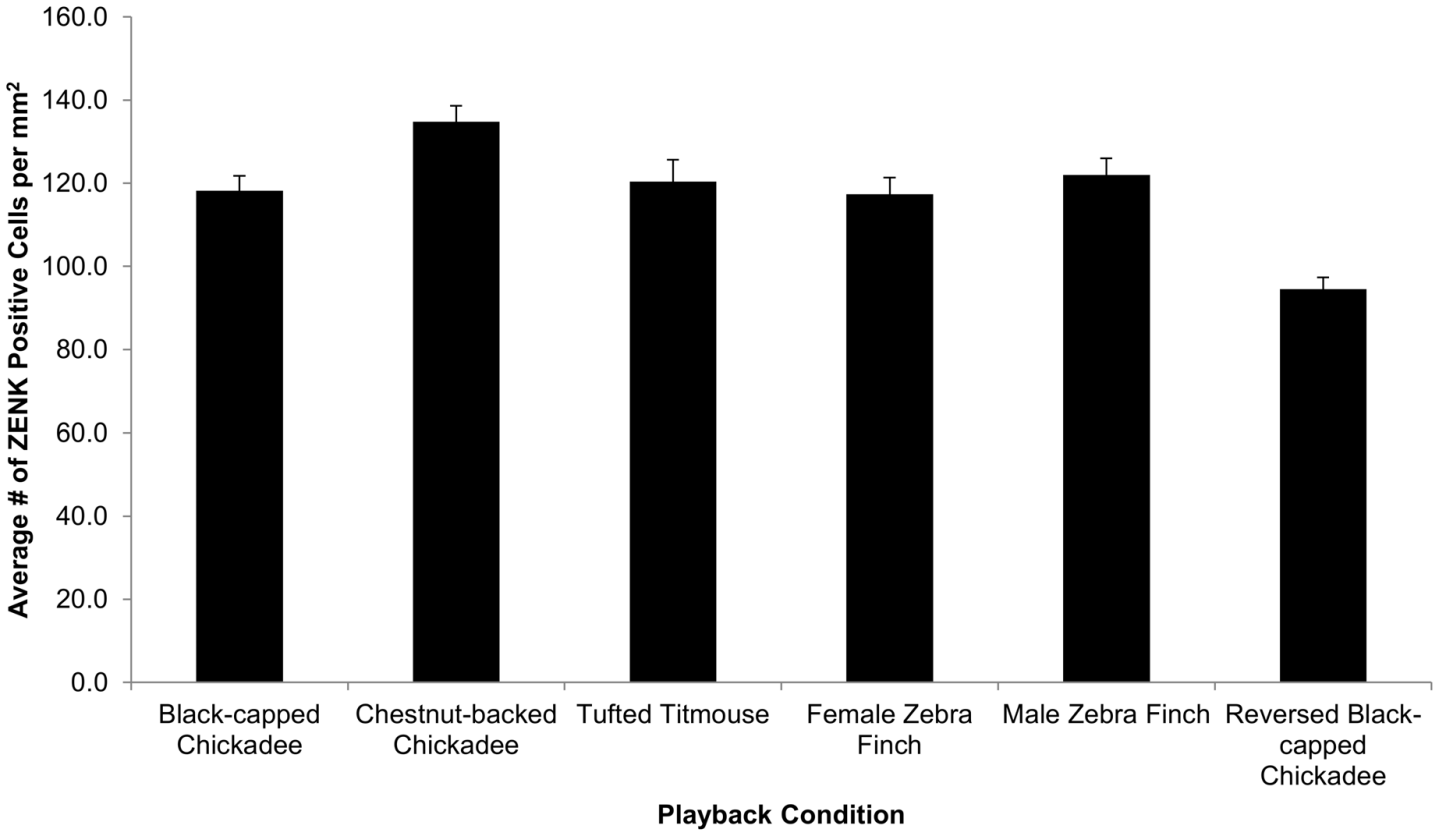
ZENK expression by playback condition. Playback condition was significant *p*<0.01, and post hoc comparisons (Tukey HSD) indicated no significant differences in mean ZENK expression between any of the playback conditions except for the reversed black-capped chickadee condition had less ZENK expression compared to all other playback conditions (black-capped chickadee, *p* = 0.04; chestnut-backed chickadee, *p*<0.01; tufted titmouse, *p* = 0.03; female zebra finch, *p* = 0.05; male zebra finch, *p* = 0.01). Y  =  mean over subjects ± SEM and X  =  Playback Condition.

**Figure 4:**
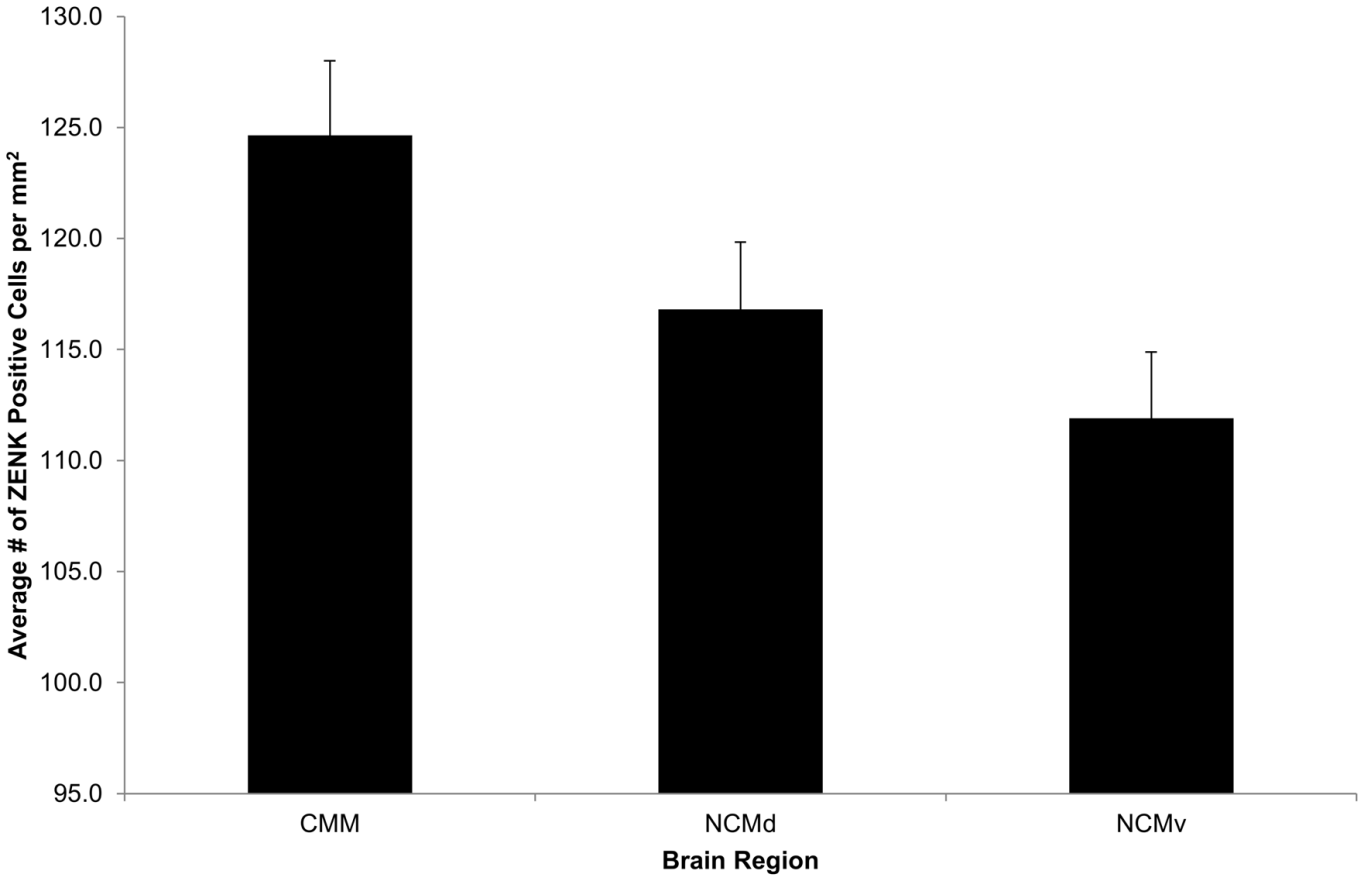
ZENK expression by brain region. There was a significant effect of brain regions on ZENK expression (*p*<0.01). Pairwise comparisons showed that this effect is due to the significant difference of expression level between the caudomedial mesopallium (CMM) and the dorsal caudal medial nidopallium (NCMd). NCMv: ventral caudal medial nidopallium. Y  =  mean over subjects ± SEM, and X  =  Brain Region.

We observed ZENK expression in all six experimental conditions with a robust response in both CMM and NCM (Fig. 2.). The repeated measures ANOVA revealed significant main effects for Playback Condition (*F* (5, 23) = 6.28, *p*<0.01; Table 2; Fig. 3.), and Brain Region (*F* (2, 46) = 6.77, *p*<0.01; Table 3; Fig. 4.). The pairwise comparisons for Brain Region revealed that CMM had significantly more ZENK expression than NCMv (*p*<0.01) but the amount of expression was not significantly different from NCMd. There was no significant difference between the two hemispheres (*F* (1, 23) = 0.03, *p* = 0.86), no significant interaction between Playback Condition and Brain Region (*p* = 0.31), and no other significant interaction terms.

**Table 2:** Mean and Standard Error of the Mean of the Number of Positive ZENK Cells Across All Brain Regions for Playback Condition.

Playback Condition	MEAN	SEM
Black-capped Chickadee	118.2	3.6
Chestnut-back Chickadee	134.8	3.9
Tufted Titmouse	120.4	5.3
Female Zebra Finch	117.4	4.0
Male Zebra Finch	122.0	4.0
Reversed Black-capped Chickadee	94.5	2.8

**Table 3:** Mean and Standard Error of the Mean of the Number of Cells for CMM, NCMd, and NCMv.

Brain Region	MEAN	SEM
CMM	124.6	3.4
NCMd	116.8	3.0
NCMv	111.9	3.0

Post hoc comparisons (Tukey HSD) for the between subject factor, Playback Condition, revealed no significant differences in mean ZENK expression among any of the playback conditions except for reversed black-capped chickadee ‘*dee*’ notes (Fig. 3.). The reversed black-capped chickadee condition had less ZENK expression compared to all other playback conditions (black-capped chickadee, *p* = 0.04; chestnut-backed chickadee, *p*<0.01; tufted titmouse, *p* = 0.03; female zebra finch, *p* = 0.05; male zebra finch, *p* = 0.01).

## Discussion

We detected no significant difference in the amount of ZENK expression regardless of whether the vocalizations were conspecific or heterospecific. These results suggest that the amount of ZENK expression becomes indistinguishable when conspecific and heterospecific calls are acoustically similar to one another. We found little ZENK expression in chickadees exposed to the reversed *‘dee’* call playbacks, indicating that the fine-scale acoustic features of the signals driving the neural response are lost or minimized when the signals are reversed. We suspect this result stems from the relatively rapid onsets and more gradual offsets of normally-produced *‘dee’* notes or distance calls irrespective of species, although further research is needed to test this possibility.

### Relationship Between Bioacoustics and Responsiveness to Heterospecific Stimuli

We used the acoustically complex *‘dee’* notes from the *chick-a-dee* calls of two chickadee species and acoustically-similar calls from the related tufted titmouse as well as acoustically-similar calls from the more distantly related zebra finch. Relative to previous research (see Table 4.), the calls used were much more similar acoustically (e.g. max frequency) and were selected to have equivalent durations (except female zebra finch calls, which were longer). Our results suggest that, when chickadees are presented with heterospecific vocalizations that have acoustic properties similar to the properties of conspecific vocalizations, then the amount of ZENK expression in CMM and NCM no longer reliably indicates differences in response between conspecific and heterospecific vocalizations.

**Table 4:** Behavioural Neuroscience Research Articles That Use Vocal Stimuli from Heterospecific Species.

Article	Species Studied	Vocal Learner	Stimulus	Technique†
Brenowitz, 1991 [60]	canary	Yes	CON, HET (white-crowned sparrow)	Lesion
Mello et al. 1992 [9]	zebra finch & canary	Yes	CON, HET (zebra finch & canary), TON, SIL	ZENK
Chew et al. 1995 [5]	zebra finch	Yes	CON, HET (canary, human words), TON, WN	EP
Chew et al. 1996 [6]	zebra finch	Yes	BOS, CON, NOV, HET (canary, Bengalese finch, silver bill, human speech) TON, WN	EP
Stripling et al. 1997 [56]	zebra finch	Yes	BOS, CON, HET (white-crowned sparrow), TON, WN, SIL	EP
MacDougall-Shackleton et al. 1998 [61]	zebra finch	Yes	CON, Artificial CON, HET (European nightingale)	Lesion
Scharff et al. 1998 [62]	zebra finch	Yes	CON, HET (canary)	Lesion
Bentley et al. 2000 [63]	canary & song sparrow	Yes	CON, HET (zebra finch), SIL	Behaviour
Rosen and Mooney, 2000 [64]	zebra finch	Yes	BOS, Reversed BOS, Reversed Syllable BOS, CON, HET (Bengalese finch), WN	EP
Mooney et al. 2001 [65]	swamp sparrow	Yes	BOS, NOV, HET (song sparrows)	EP
Stripling et al. 2001 [10]	zebra finch	Yes	CON, Reversed CON, NOV, HET (white-crowned sparrow), TON, WN	EP
Bailey et al. 2002 [66]	zebra finch	Yes	CON, HET (*), TON, SIL	ZENK
Long et al. 2002 [67]	chicken & quail	No	CON, HET (chicken or quail)	ZENK
Bailey and Wade, 2003 [47]	zebra finch	Yes	CON, HET (**), TON, SIL	FOS
Hernandez and MacDougall-Shackleton, 2004 [8]	house finch	Yes	Local NOV, Foreign NOV, HET (white-crowned sparrow)	ZENK
Bailey and Wade, 2005 [48]	zebra finch	Yes	Female directed NOV, HET (**), SIL	ZENK
Terpstra et al. 2005 [68]	ringdove	No	NOV, HET (zebra finch)	ZENK
Velho et al. 2005 [11]	zebra finch	Yes	NOV, HET (canary), TON, WN, SIL	ZENK
Bailey and Wade., 2006 [69]	zebra finch	Yes	CON, HET (**), TON, SIL	FOS, ZENK
Huchzermeyer et al. 2006 [70]	zebra finch	Yes	CON, HET (Bengalese finch)	ZENK
Lynch and Ball, 2008 [7]	canary	Yes	NOV, HET (Cassin's finch)	ZENK
Gee et al. 2009 [71]	California & Gambel's quail	No	NOV, HET (California or Gambel's quail), TON	ZENK
Poirier et al. 2009 [43]	zebra finch	Yes	BOS, CON, HET (canary or starling)	fMRI
Avey et al. 2011 [37]	black-capped & mountain chickadee	Yes	CON, HET (***)	ZENK
Phillmore et al. 2011 [49]	black-capped chickadee	Yes	CON, HET (song sparrow)	ZENK
Poirier et al. 2011 [72]	zebra finch	Yes	BOS, CON, HET (canary or starling)	fMRI
Yoder et al. 2012 [73]	zebra finch	Yes	CON, HET (canary)	EP

† Lesions; ZENK/FOS, immediate early gene labeling for ZENK, C-FOS; EP, electrophysiology; Behaviour, behavioural studies; fMRI, functional magnetic resonance imaging.

Songbird Species: canary, house finch, song sparrow, swamp sparrow, zebra finch.

Non-Songbird Species: chicken, California quail, Gambel's quail, quail, ringdove.

Stimulus: Bird's Own Song (BOS), Conspecific (CON), Novel Conspecific (NOV), Heterospecific (HET (species)), Tone (TON), Silence (SIL), White Noise (WN).

*American robin, summer tanager, Bell's vireo, white breasted nuthatch, marsh wren, Connecticut warbler, cassin's finch, Baird's sparrow, Scott's oriole, western meadowlark.

**American robin, Baird's sparrow, Bell's vireo, Cassin's finch, Connecticut warbler, marsh wren, Scott's oriole, summer tanager, western meadowlark, and white-breasted nuthatch.

***Black-capped & mountain chickadee, great-horned owl, northern saw-whet owl, red-breasted nuthatch.

These patterns of activation may reflect that secondary auditory areas of chickadees are mainly processing the fine acoustic properties of the stimuli. The reversed black-capped ‘*dee*’ notes resulted in significantly less ZENK expression than the other playback conditions although the absolute amount of expression to these reversed ‘*dee*’ notes was still high. The reduction in ZENK expression to reversed ‘*dee*’ notes is in line with previous behavioural findings that playbacks of whole *chick-a-dee* calls (i.e. abc-‘dee’) that have been reversed (i.e. ‘*dee*’-cba) induced less calling behaviour from black-capped chickadees than other control stimuli (i.e. gray-crowned rosy-finch calls) [41]. Which particular acoustic features of the reversed black-capped ‘*dee*’ notes caused this difference is unclear, but future behavioural testing via field playback and operant discrimination paradigms can further explore these acoustic features.

Black-capped chickadees are able to discriminate between conspecific and heterospecific species using acoustic features of the ‘*dee*’ note [27], [29], [30], [42]. However, our results suggest that ZENK expression alone as a neural activity marker, quantified and compared via the number of ZENK-positive cells in CMM and NCM, cannot explain the neural mechanism for species discrimination using the acoustic features of the ‘*dee*’ note only. Neural decoding for species recognition may occur outside of these brain regions (e.g. MLd; [43]), or different neuronal populations may encode for different relevant stimuli. For instance, ZENK expression for conspecific stimuli may be induced in a different set of neurons than for heterospecific stimuli. Thus, there may be distinct neurons for different stimuli (i.e. selectivity), although the overall number of neurons may be similar. Alternatively, the neural activity as measured by ZENK expression may not be capturing subtle changes in neural electrophysiological activity. Future studies using *in vivo* electrophysiological paradigms should investigate if cells in these regions are selective for conspecific stimuli and/or heterospecific stimuli.

Outside CMM and NCM there is evidence that the lateral mesencephalic nucleus (MLd) may partially account for species discrimination [43]. Two alternate hypotheses both suggest that neural selectivity for the bird's own song (BOS) may explain the ability to differentiate between conspecific and heterospecific vocalizations. One hypothesis suggests that the neural selectivity for BOS may explain the ability of birds to identify conspecific song and thus could also explain the ability to discriminate between conspecific and heterospecific song [43], [44]. Another hypothesis is that there are distinct neural substrate for BOS and conspecific song recognition with evidence that BOS recognition involves the right MLd and species recognition involves the left MLd [43].

### CMM and NCM's Responsiveness to Conspecific and Heterospecific Stimuli

The available evidence suggests CMM and NCM play a role in the processing of heterospecific signals, including the ability to discriminate between relevant conspecific and heterospecific vocalizations. Generally, both CMM and NCM have similar activation patterns in response to conspecific and heterospecific stimuli, with conspecific stimuli generating a greater response than heterospecific stimuli [9], [10] and it has been suggested that the expression is related to stimulus type and familiarity [45]. Numerous studies have found that conspecific stimuli generate more immediate early gene expression, neural electrophysiological activity, or BOLD activity using a variety of paradigms (see Table 4.).

Nevertheless, previous studies (see Table 4.) did not select heterospecific stimuli from closely related species or with similar acoustic properties. The resulting sharp differences in the acoustic properties of the stimuli could explain the consistent difference between conspecific and heterospecific ZENK expression in the formerly mentioned studies as well as in other studies. The results from the current experiment strongly suggest that as the acoustic properties converge between conspecific and heterospecific vocalizations, differences in the amount of ZENK expression disappear altogether in CMM and NCM. This activation pattern supports the idea that the auditory forebrain is preferentially responsive to conspecific and heterospecific signals with similar acoustical structures [46]. One consequence is that heterospecific vocalizations should be carefully chosen when used as control stimuli in behavioural and neurophysiological experiments investigating the brain's selectivity for conspecific vocalizations. On one hand, acoustically divergent heterospecific stimuli might be salient stimuli but they do not control for the difference of acoustics that could be driving the responsiveness *per se*. On the other hand, acoustically similar heterospecific stimuli may be unsuitable as a control condition because there will be no difference in responsiveness.

Some studies have also found no difference in neural activity between conspecific and heterospecific stimuli. First, this contrasted response seems to be developed at different ages in juveniles depending on their sex. In juvenile (day 30) female zebra finches there is no difference in expression of ZENK (and c-FOS) between birds exposed to conspecific stimuli and birds exposed to heterospecific stimuli; although a difference in expression is detectable in juvenile male zebra finches [47]. By day 45 however, the expression to conspecific stimuli is greater than heterospecific stimuli in both sexes of zebra finches as in adults [48]. Second, this contrasted response may be the result of the salience of the signal. In black-capped chickadees exposed to either black-capped or mountain chickadee (*Poecile gambeli*; sister species) mobbing calls there was no difference between ZENK expression in either CMM or NCM, and exposure to high threat predator vocalizations resulted in greater ZENK expression than to conspecific low threat mobbing calls [37]. ZENK expression in response to heterospecific stimuli also varies with season with relatively more expression in black-capped chickadees in the non-breeding condition than in the breeding condition [49].

### Spatial Distribution of Responsiveness to Heterospecific Stimuli

In line with previous studies with black-capped chickadees we did not find any significant hemispheric difference in the response of CMM and NCM to conspecific or heterospecific stimuli [26], [37]. However previous study using zebra finches and European starlings (*Sturnus vulgaris*) found evidence that auditory processing may be lateralized [36], [50]. For instance, zebra finches exposed to both auditory and visual cues show a lateralization of ZENK expression in these brain regions [35]. Using fMRI, lateralization in NCM has been found in European starlings listening to conspecific signals [51]. Taken together this evidence suggests that the processing of auditory stimuli is lateralized but what features drive this response remain unknown and appear to be unrelated to the difference between conspecific and heterospecific stimuli.

### Relevance of Responsiveness to Heterospecific Stimuli

Why might there be such strong neural activation to conspecific *‘dee’* notes as well as to heterospecific *‘dee’* notes and to acoustically-similar notes of distantly-related species? The *chick-a-dee* call of parid species is used in a wide range of contexts related to social cohesion [52]. The *‘dee’* note of the call, in particular, seems to be produced more by signalers facing situations of increased arousal or threat [52]. Playback studies manipulating number of *‘dee’* notes in calls [53]–[55] or overall number of *‘dee’* notes played back [56], [57] reveal that stimuli with more *‘dee’* notes elicit faster recruitment of flock members to the playback area and greater mobbing-like behavior of individuals already in the playback area. Given the contexts of greater production of *‘dee’* notes – detection of food [54] and predators [58], [59] – there should be strong selection pressure on the part of receivers to attend to these notes when they are produced by signalers. Furthermore, many parid species occur in relatively stable mixed-species flocks that include other parids, and so it may be adaptive to respond to calls of other parid species. As such, there should also be neurophysiological properties to process rapidly such sounds in these species and tolerate some distortion of the signal. If so, any sound with *‘dee’*-like acoustic properties may be a biologically-meaningful stimulus to individuals of these species, with the result that neural regions like CMM and NCM show invariant responses and are activated by *‘dee’*-like stimuli, regardless of the evolutionary relatedness of the source of the sound.

### Conclusion

In conclusion, we show that the level of activity in secondary auditory areas of black-capped chickadees, as revealed by the immediate early gene ZENK, is not always greater in response to conspecific signals than in response to heterospecific signals. When acoustic properties of heterospecific and conspecific vocalizations are similar, the number of cells expressing ZENK in CMM and NCM is similar. Thus, differences in the number of ZENK immunoreactive cells in secondary auditory areas would be predictive of acoustic dissimilarities between stimuli, and not necessarily of species discrimination.
